# Interpretable machine learning for predicting 30-day mortality following intracranial hemorrhage surgery

**DOI:** 10.3389/fneur.2026.1716346

**Published:** 2026-02-13

**Authors:** Ziyang Wang, Wenbin Chen, Yan Shi

**Affiliations:** Anesthesiology, Ningde Municipal Hospital of Ningde Normal University, Ningde, China

**Keywords:** intracranial hemorrhage, lactate, mortality, prediction, XGBoost

## Abstract

**Objective:**

This study aims to utilize interpretable machine learning models based on perioperative data to forecast the 30-day mortality risk following intracranial hemorrhage (ICH) surgery. By employing SHapley Additive exPlanations (SHAP) to interpret the Extreme Gradient Boosting (XGBoost) model, we sought to identify modifiable prognostic factors to improve clinical decision-making.

**Methods:**

A retrospective analysis was conducted on perioperative data from 1,271 ICH patients. After applying exclusion criteria, 992 patients were included. The dataset was randomly partitioned into training and validation cohorts (7:3 ratio). Multiple machine learning algorithms, including logistic regression, SVM, Random Forest, and XGBoost were developed. Model performance was rigorously assessed via ROC curves, calibration curves, and decision curve analysis (DCA), with hyperparameters optimized using 5-fold cross-validation.

**Results:**

The observed 30-day postoperative mortality rate was 13%. The XGBoost model achieved an AUC of 0.931 (95% CI 0.91–0.96) in the training cohort and 0.937 (95% CI 0.90–0.97) in the validation cohort, outperforming the logistic regression model (AUC 0.669). Decision curve analysis indicated that the XGBoost model provided the greatest net benefit within a threshold probability range of 5.79 to 33.52%. SHAP analysis identified postoperative pH, lactate, APTT, and CRP as the primary predictive factors.

**Conclusion:**

This study establishes an interpretable XGBoost model that leverages perioperative data to accurately predict short-term mortality after ICH surgery. By highlighting the prognostic value of these modifiable biomarkers, the model serves as a practical tool for early risk stratification, assisting in the optimization of perioperative management in critical care settings.

## 1 Introduction

Intracerebral hemorrhage (ICH) is a critical cerebrovascular event marked by significant morbidity, disability, and mortality rates (1). According to the latest epidemiological data (2), millions of new ICH cases occur globally each year, imposing a substantial physical and psychological burden on patients and their families. ICH not only leads to serious neurological dysfunctions, such as motor, speech, and cognitive impairments, but also is often accompanied by complications like cerebral edema (3), hematoma expansion (4), and increased intracranial pressure, further complicating and challenging treatment (5).

Current prognostic tools for ICH have made significant strides, yet many gaps remain. Traditional models like the ICH score and FUNC score have been widely used but often show limitations, particularly in more complex clinical cases (6). A meta-analysis by Gregório et al. (7) in 2018 showed that while these regression-based models provide reasonable discrimination, their calibration is often suboptimal. Additionally, existing machine learning approaches, such as those by Xu et al. (8) and Nie et al. (9), have attempted to overcome these challenges by utilizing advanced algorithms like Random Forest and LightGBM, which better capture the non-linear relationships between clinical variables (10, 11). These models have shown promising results, improving accuracy in predicting favorable or unfavorable outcomes with higher AUC scores and more accurate risk predictions (12).

However, these models have not fully considered intraoperative and postoperative factors that can significantly influence patient outcomes (13). Crucial variables such as anesthesia management, intraoperative blood pressure control, and surgical interventions are often overlooked in current predictive models, despite their critical importance in shaping recovery. Furthermore, many studies rely on relatively small sample sizes, which reduces the statistical power of the results and limits their applicability to broader populations. Open critical care database.

Addressing these limitations, this study aims to develop a comprehensive machine learning-based model to predict the short-term prognosis of patients with ICH, utilizing a publicly perioperative database—the INSPIRE dataset. Additionally, the SHapley Additive exPlanations (SHAP) method will be employed to interpret the XGBoost model, identifying key prognostic factors that influence patient outcomes and enhancing the model’s clinical applicability.

## 2 Methods

### 2.1 Data source

This retrospective analysis utilized perioperative data from the INSPIRE database (version 1.1), a comprehensive perioperative medical database maintained by a South Korean academic institution (14). The INSPIRE database, spanning from 2011 to 2020, includes detailed medical records of approximately 130,000 patients who underwent surgery under anesthesia, providing an extensive source of high-quality perioperative information.

### 2.2 Ethical considerations

Ethical approval and patient consent were unnecessary as all health information was anonymized.

### 2.3 Study population

The research focused on patients diagnosed with intracerebral hemorrhage as per ICD-10 codes. A total of 1,271 patients were initially identified. Among these, 1,142 cases were recorded as the primary diagnosis upon admission. Of these, 1,066 patients underwent craniotomy surgery.

The study cohort was refined using specific inclusion and exclusion criteria. The study excluded patients with over 15% missing data (*n* = 52) and those under 18 years old (*n* = 22), resulting in 992 patients meeting the inclusion criteria for the final analysis.

### 2.4 Predictor variables

(1) Demographics: including variables such as age, sex, and body mass index (BMI); (2) Comorbidities: covering conditions like hypertension, diabetes, atrial fibrillation, coronary artery disease, chronic heart failure, chronic obstructive pulmonary disease (COPD), renal insufficiency, prior stroke (hemorrhagic or ischemic), and cancer; (3) Medications: including the use of β-blockers, ACE inhibitors/angiotensin receptor blockers (ACEI/ARB), statins, calcium channel blockers (CCB), insulin, glucocorticoids, anticoagulants, and antiplatelet drugs; (4) Surgical Details: including ASA classification, intraoperative events such as hypotension, bradycardia, tachycardia, and operation time; (5) Laboratory Tests: such as hemoglobin, platelets, APTT, AST, ALT, albumin, hs-CRP, lactate, WBC, pH, PaO2, and creatinine levels, which were collected during the first examination upon admission. Vital signs during surgery were recorded every 5 min. Intraoperative hypotension (IOH) was defined as a mean arterial pressure less than 65 mmHg or a decrease greater than 20% from the preoperative value for two consecutive readings (15). Intraoperative bradycardia was defined as a heart rate less than 50 bpm for two consecutive measurements.

### 2.5 Missing data handling

Handling missing data is a frequent challenge when working with the INSPIRE open database. Ignoring missing data can lead to biased results, which could compromise the validity of the study. To address this issue, we applied multiple imputation to fill in missing values (16). All selected variables had fewer than 15% of their data missing. Assuming that the data were missing at random, we employed the fully conditional specification method to impute the missing data, using the “mice” package (version 3.13.0) in R (version 4.1.0; R Core Team) (16).

### 2.6 Machine learning explainability tool

The SHAP is employed to interpret the prediction model by accurately determining each feature’s contribution and impact on the final predictions. SHAP values quantify each predictor’s positive or negative contribution to the target variable (16). Additionally, each observation in the dataset can be analyzed using its specific set of SHAP values.

### 2.7 Statistical analysis

All statistical analyses and computations were performed using R software and Python (version 3.8.0; Python Software Foundation). Categorical variables were expressed as counts and percentages, and group differences were evaluated using the *χ*^2^ test or Fisher’s exact test when expected frequencies were less than 10. Continuous variables were represented as medians with interquartile ranges (IQR) and compared across groups using the Wilcoxon rank-sum test.

To ensure the internal validity of the algorithms, the entire dataset was randomly partitioned into a training cohort and a validation cohort at a ratio of 7:3. The training cohort was utilized to train the algorithms and optimize hyperparameters using 5-fold cross-validation grid search to prevent overfitting. Subsequently, the validation cohort served as an independent dataset for performance evaluation. Predictive models were developed using four machine learning techniques: XGBoost, logistic regression (LR), random forest (RF), and support vector machine (SVM). Each model’s predictive performance was assessed via the area under the receiver operating characteristic curve (AUC). We determined accuracy, sensitivity, positive predictive value (PPV), negative predictive value (NPV), and the F1 score with predictive specificity set at 85%. Decision curve analysis (DCA) was performed to evaluate model utility (a method that quantifies the net clinical benefit by balancing overtreatment risks and missed diagnosis costs across probability thresholds, aiming to assess the practical value of predictive models in real-world clinical decision-making) by measuring net benefits at various probability thresholds (17, 18).

## 3 Results

### 3.1 Patient characteristics

**Figure 1:**
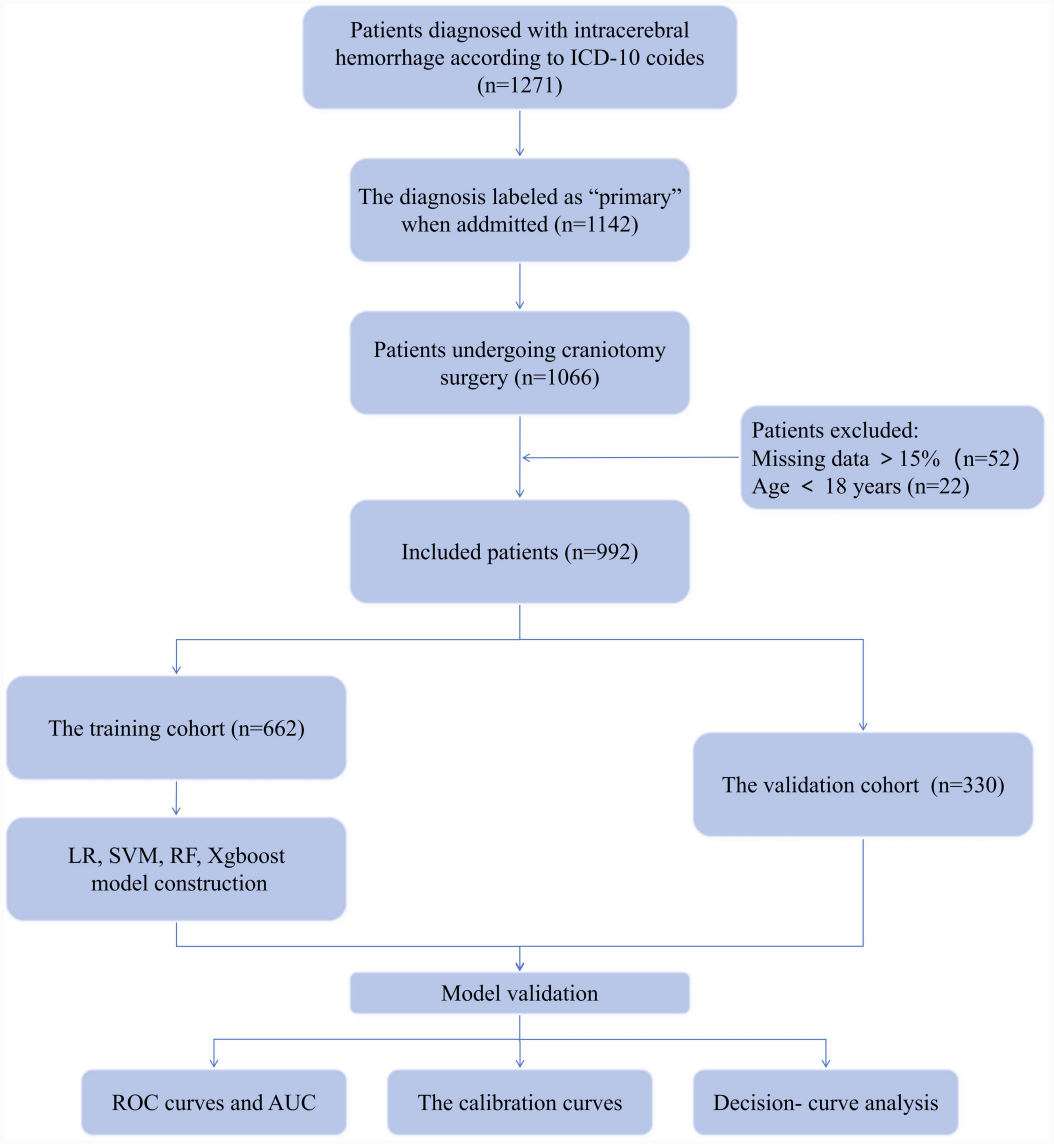
Flow chart for patient selection. LR, logistic regression; RF, random forest; SVM, support vector machine.

This study included 1,271 patients diagnosed with ICH according to ICD-10 coding. Upon admission, 1,142 patients had a primary diagnosis of ICH, and 1,066 of these patients underwent craniotomy. After excluding 52 patients with more than 15% missing data and 22 patients under the age of 18, 992 patients were included for analysis. Figure 1 illustrates the patient screening process. The patients were allocated into a training cohort of 662 individuals and a validation cohort of 330 individuals. The mortality group had significantly more males than females (*p* < 0.05) (Table 1).

**Table 1:** Characteristics and outcomes of patients undergoing craniotomy surgery (*n* = 992).

Characteristic	Survivors (*n* = 863)	Non-survivors (*n* = 129)	*χ*^2^/*F*	*P*-value
Demographics				
Age (yr), median (IQR)	65.00 (55.00–75.00)	70.00 (55.00–71.25)	0.010	0.922
Male, *n* (%)	484 (56.08)	90 (69.77)	4.708	0.03
BMI (kg/m^2^), median (IQR)	22.37 (19.63–24.72)	22.55 (20.32–22.98)	0.160	0.689
Comorbidities, *n* (%)				
Hypertension	196 (22.71)	32 (24.81)	0.031	0.860
Diabetes	109 (12.64)	39 (30.23)	2.471	0.116
Atrial fibrillation	65 (7.53)	13 (10.08)	0.023	0.879
Coronary artery disease	62 (7.18)	25 (19.38)	2.752	0.097
Chronic heart failure	22 (2.55)	0 (0.00)	0.613	0.434
COPD	16 (1.85)	0 (0.00)	0.457	0.499
Renal insufficiency	41 (4.75)	19 (14.77)	2.139	0.144
Prior hemorrhagic stroke	197 (22.82)	39 (30.23)	0.023	0.879
Prior ischemic stroke	107 (12.39)	32 (24.81)	0.886	0.346
Cancer	112 (12.95)	32 (24.81)	0.718	0.397
Surgical conditions				
ASA classification, *n* (%)			2.331	0.675
<3	357 (41.37)	48 (37.21)		
≧3	506 (58.63)	81 (62.79%)		
Intraoperative hypotension, *n* (%)	28 (3.24)	2 (1.55%)	0.174	0.676
Intraoperative bradycardia, *n* (%)	7 (0.81)	0 (0.00%)	1.095	0.295
Intraoperative tachycardia, *n* (%)	87 (10.09)	3 (2.33%)	2.943	0.086
Operation time (min), median (IQR)	125.00 (81.25–185.00)	147.50 (112.50–197.50)	1.850	0.174
Use of medications, *n* (%)				
β-blocker	134 (15.52)	8 (6.20%)	84.390	1.000
ACEI/ARB	125 (14.48)	8 (6.20%)	1.427	0.232
Statin	108 (12.52)	6 (4.65%)	0.339	0.560
CCB	124 (14.38)	8 (6.20%)	1.596	0.206
Insulin	134 (15.52)	8 (6.20%)	84.390	1.000
Glucocorticoid	97 (11.25)	4 (3.10%)	4.113	0.043
Tranexamic acid	125 (14.48)	8 (6.20%)	1.427	0.232
Anticoagulant	97 (11.25)	4 (3.10%)	4.113	0.043
Antiplatelet drug	116 (13.43)	6 (4.65%)	0.613	0.434
Norepinephrine	108 (12.52)	6 (4.65%)	0.339	0.560
Epinephrine	0 (0.00%)	3 (0.35)	0.457	0.499
Dopamine	131 (15.17)	8 (6.20%)	0.457	0.499
Laboratory tests, median (IQR)				
Hemoglobin (g/dl)	10.90 (9.40–11.20)	9.40 (8.60–10.98)	1.475	0.225
Platelets (10^9^/l)	231.00 (209.00–255.22)	231.00 (220.00–231.00)	0.542	0.462
APTT (s)	30.54 (27.90–33.00)	30.62 (28.35–34.80)	1.361	0.243
AST (u/l)	19.00 (16.00–20.00)	18.50 (17.50–20.75)	0.361	0.548
ALT (u/l)	15.60 (9.00–22.00)	15.80 (9.00–22.00)	0.591	0.442
Albumin (g/dl)	3.20 (3.10–3.27)	3.21 (3.10–3.60)	1.189	0.275
Hs-CRP (mg/l)	2.25 (0.50–3.86)	2.04 (0.31–2.94)	0.391	0.532
Lactate (mmol/l)	1.20 (0.50–2.80)	2.20 (1.20–3.00)	3.549	0.060
WBC (10^9^/l)	7.00 (6.62–7.84)	7.00 (6.91–8.43)	0.083	0.773
PH	7.37 (7.37–7.38)	7.37 (7.34–7.37)	2.236	0.135
PaO2 (mmHg)	148.00 (125.00–148.00)	130.40 (125.00–148.00)	0.407	0.524
Creatinine (mg/dl)	0.85 (0.66–0.89)	0.71 (0.62–0.85)	3.365	0.067
Outcomes				
ICU admission, *n* (%)	751 (87.02)	123 (95.35)	0.996	0.318
Length of stay (day)	21.00 (14.00–48.50)	47.50 (18.00–58.00)	1.836	0.175

BMI, body mass index; CCB, calcium channel blocker; COPD, chronic obstructive pulmonary disease; ICU, intensive care unit; IQR, interquartile range; APTT, activated partial thromboplastin time; AST, aspartate aminotransferase; ALT, alanine aminotransferase; Hs-CRP, high-sensitivity C-reactive protein; WBC, white blood cell; PH, potential of hydrogen; PaO2, partial pressure of oxygen; ASA, American Society of Anesthesiologists; ACEI, angiotensin-converting enzyme inhibitor; ARB, angiotensin receptor blocker.

### 3.2 Model building and evaluation

**Figure 2:**
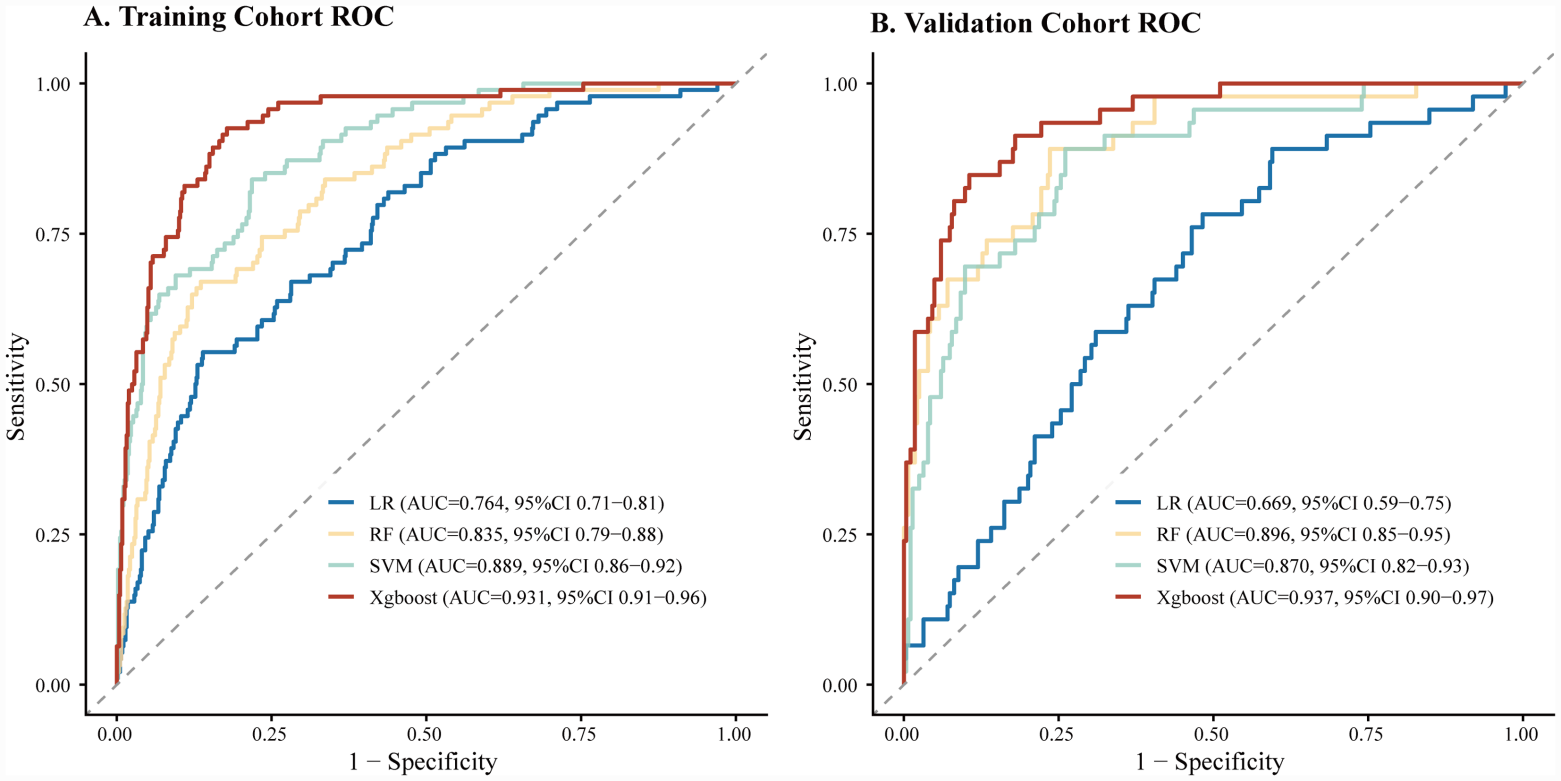
Receiver operating characteristic (ROC) curves of the four machine learning models: **(A)** ROC curves in the training cohort and **(B)** ROC curves in the validation cohort. The XGBoost model demonstrated the highest AUC values in both cohorts, indicating superior discriminative ability. AUC, area under the curve; LR, logistic regression; RF, random forest; SVM, support vector machine; XGBoost, extreme gradient boosting.

Four machine learning models (XGBoost, SVM, RF, and LR) were developed using the training cohort and comprehensively evaluated in the validation cohort. As detailed in Table 2 and Figure 2, the XGBoost model consistently demonstrated the highest predictive performance. In the training cohort, XGBoost achieved an AUC of 0.931 (95% CI: 0.91–0.96), followed by SVM (AUC: 0.889), RF (AUC: 0.835), and LR (AUC: 0.764). Consistent with the training results, the XGBoost model maintained its predictive performance in the validation cohort, achieving an AUC of 0.937 (95% CI: 0.90–0.97). In comparison, the RF and SVM models showed moderate performance with AUCs of 0.896 and 0.870, respectively, while the LR model exhibited the lowest discrimination (AUC: 0.669).

**Table 2:** Performance of each model for prediction.

Model	AUC (95% CI)	Sensitivity (%)	Specificity (%)	Accuracy (%)	PPV (%)	NPV (%)	F1 score
Training cohort							
Xgboost	0.931 (0.91–0.96)	88.5	86.4	87.2	56.8	97.5	0.692
SVM	0.889 (0.86–0.92)	82.1	83.5	83.3	48.2	96.1	0.607
RF	0.835 (0.79–0.88)	75.6	80.2	79.6	41.5	94.4	0.536
LR	0.764 (0.71–0.81)	64.2	76.8	75.1	33.7	91.8	0.442
Validation cohort							
Xgboost	0.937 (0.90–0.97)	89.2	87.1	87.4	58.1	97.8	0.704
RF	0.896 (0.85–0.95)	84.6	82.5	82.8	49.5	96.5	0.625
SVM	0.870 (0.82–0.93)	80.4	81.2	81.1	46.8	95.4	0.591
LR	0.669 (0.59–0.75)	48.5	72.4	69.2	26.3	88.1	0.341

AUC, area under the curve; PPV, positive predictive value; NPV, negative predictive value; LR, logistic regression; RF, random forest; SVM, support vector machine.

**Figure 3:**
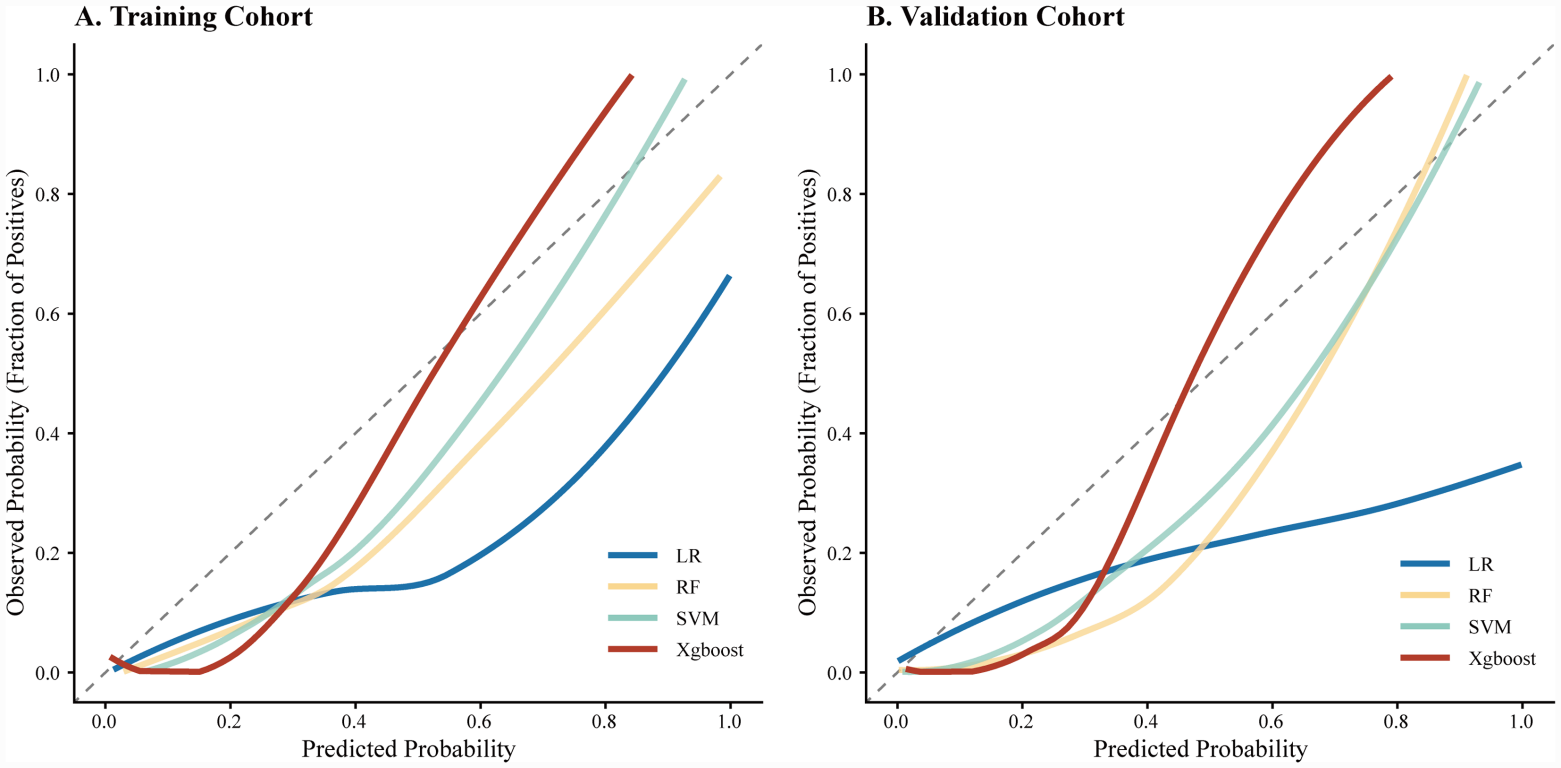
Calibration curves of the four machine learning models: **(A)** Calibration curves in the training cohort and **(B)** calibration curves in the validation cohort. The *x*-axis represents the predicted probability of 30-day mortality, and the *y*-axis represents the actual observed fraction of positives. The diagonal dashed line represents perfect prediction accuracy. LR, logistic regression; RF, random forest; SVM, support vector machine; XGBoost, extreme gradient boosting.

Regarding specific metrics in the validation cohort, the XGBoost model provided a comprehensive balance of performance, with a sensitivity of 89.2%, specificity of 87.1%, and accuracy of 87.4%. Conversely, the LR model showed limited sensitivity (48.5%) and accuracy (69.2%). The calibration curves (Figure 3) indicated that while SVM and RF showed reasonable calibration, the XGBoost model displayed the highest concordance between predicted probabilities and observed outcomes across both cohorts.

**Figure 4:**
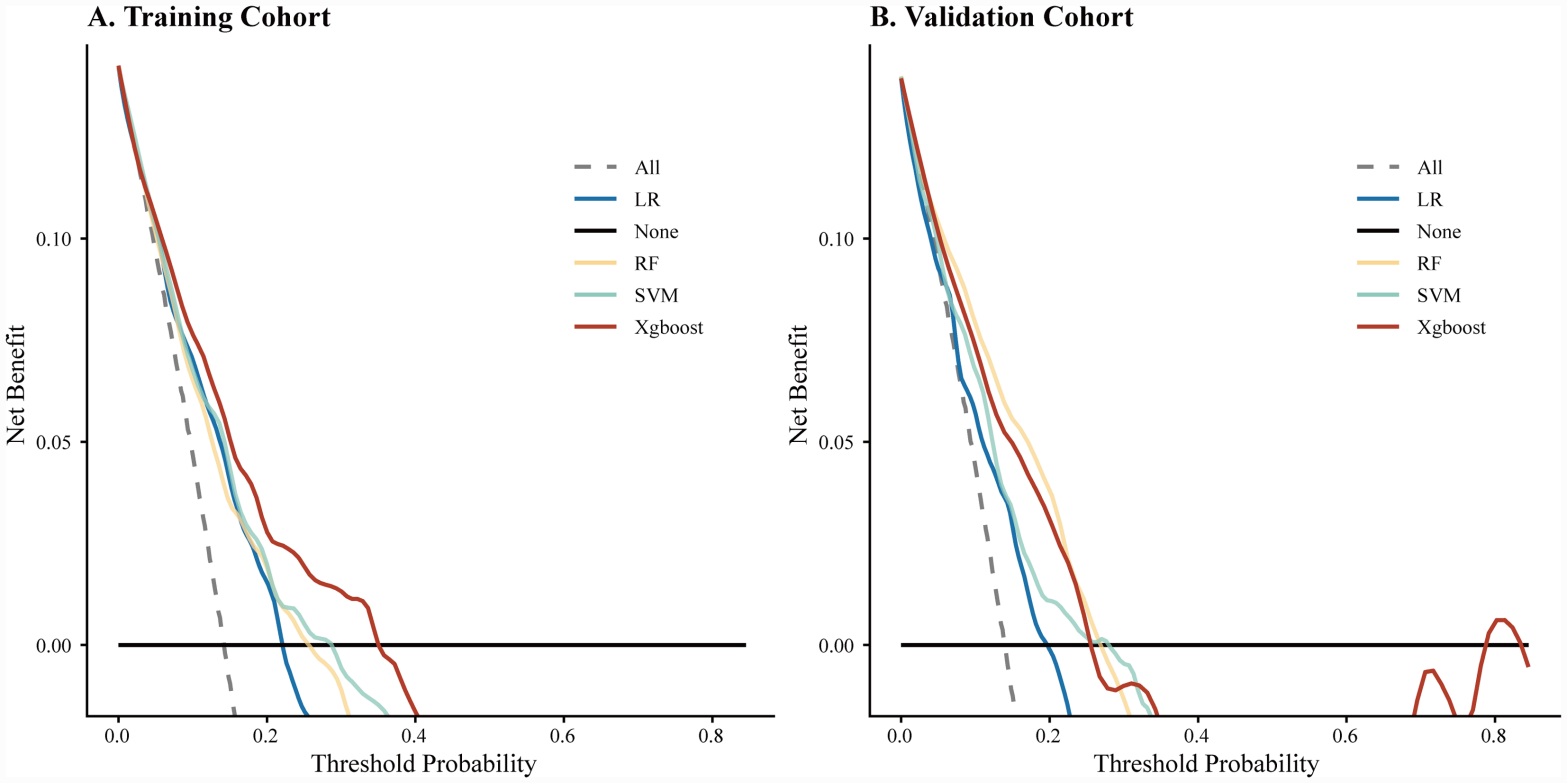
Decision curve analysis (DCA) of the four machine learning models. **(A)** DCA in the training cohort. **(B)** DCA in the validation cohort. The *y*-axis measures the net benefit. The black horizontal line represents the assumption that no patients die (Net Benefit = 0), and the gray dashed line represents the assumption that all patients die. The XGBoost model provided the greatest net benefit across the widest range of threshold probabilities. LR, logistic regression; RF, random forest; SVM, support vector machine; XGBoost, extreme gradient boosting.

The DCA further assessed the clinical utility (Figure 4). While all machine learning models offered some net benefit over default strategies, the XGBoost model provided the highest net benefit across the widest range of threshold probabilities, confirming its practical applicability compared to the other algorithms.

### 3.3 SHAP analysis of XGBoost model

**Figure 5:**
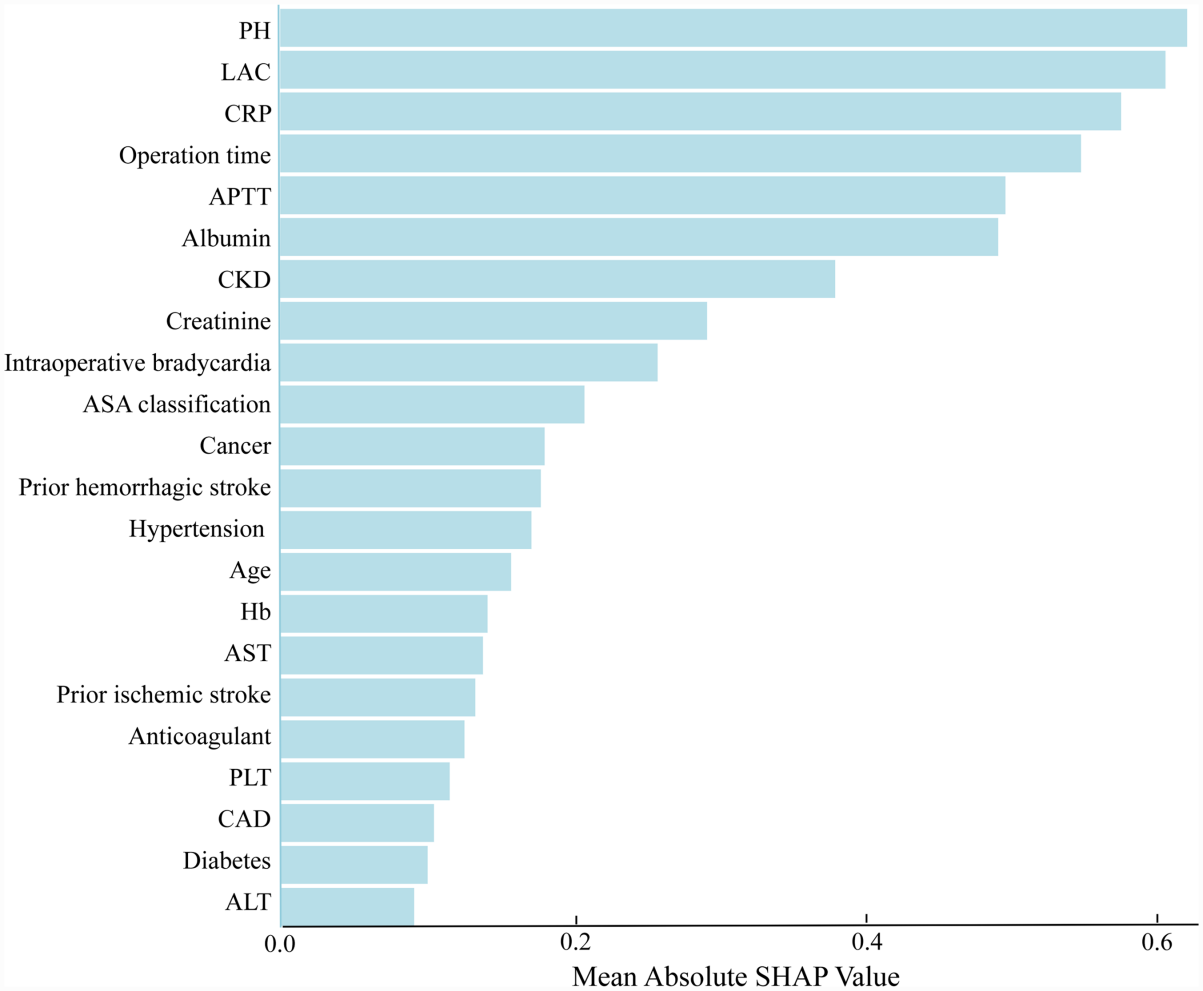
The weights of variables importance. APTT, activated partial thromboplastin time; AST, aspartate aminotransferase; ALT, alanine aminotransferase; CRP, C-reactive protein; PH, potential of hydrogen; ASA, American Society of Anesthesiologists; CKD, chronic kidney disease; Hb, hemoglobin; PLT, platelet; CAD, coronary artery disease; LAC, lactate.

**Figure 6:**
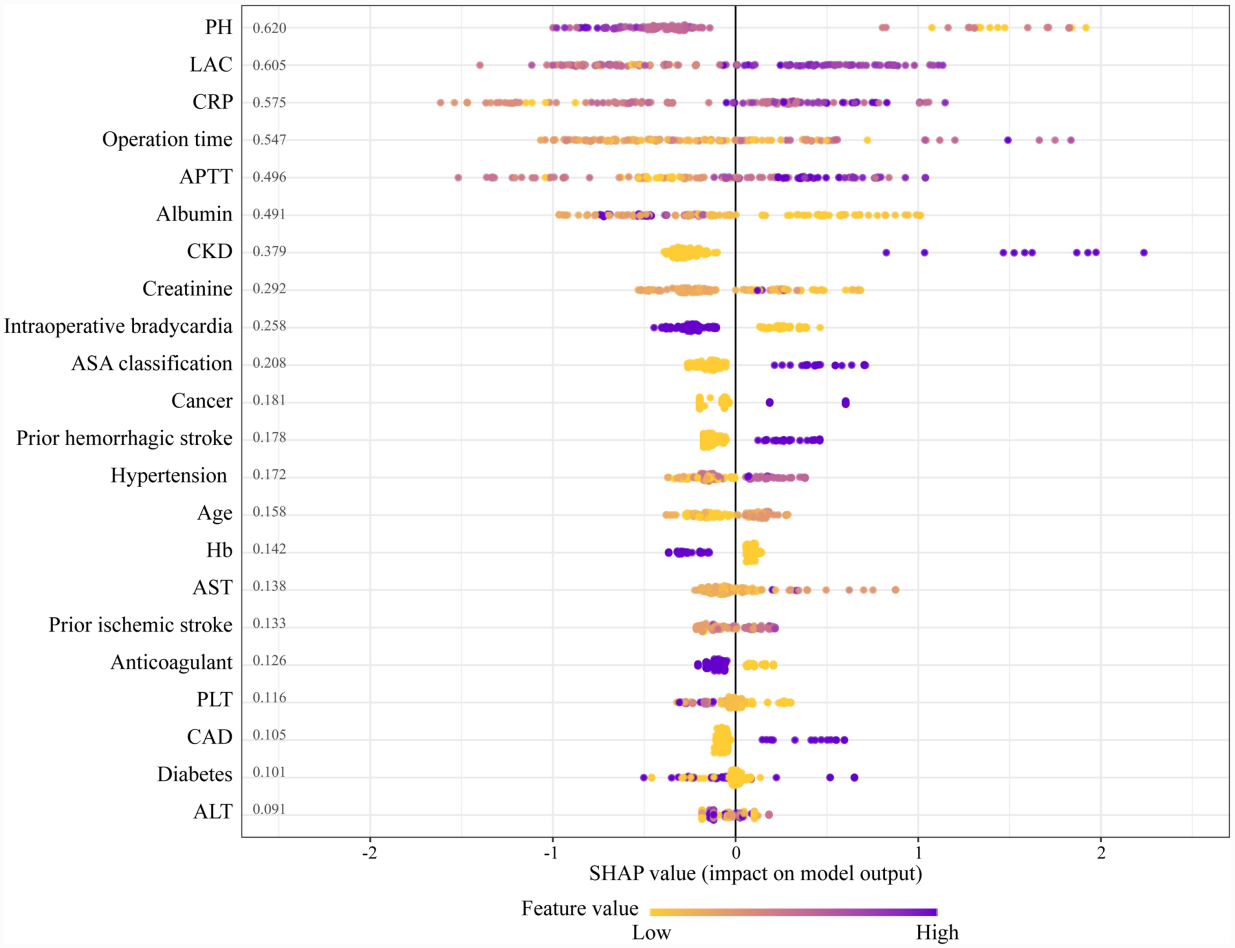
The SHapley additive explanation (SHAP) values. APTT, activated partial thromboplastin time; AST, aspartate aminotransferase; ALT, alanine aminotransferase; CRP, C-reactive protein; PH, potential of hydrogen; ASA, American Society of Anesthesiologists; CKD, chronic kidney disease; Hb, hemoglobin; PLT, platelet; CAD, coronary artery disease; LAC, lactate.

The SHAP algorithm is used to determine the importance of each predictor in the XGBoost model’s predictions. Figure 5 presents a variable importance chart that ranks the variables in order of descending importance. The mean pH value demonstrates the greatest predictive power across all ranges, with lactate, CRP, operation time, and APTT following in descending order. SHAP values are used to identify mortality risk factors by detecting positive and negative correlations between predictors and the target outcome. Figure 6 illustrates that the horizontal position reflects a value’s impact on prediction levels, with color indicating the variable’s magnitude—purple for high and yellow for low. It is evident that an increase in mean pH reduces the risk of mortality, while an increase in lactate raises the risk, steering predictions toward a higher likelihood of mortality.

## 4 Discussion

This study developed and validated a machine learning algorithm based on the XGBoost model, significantly enhancing the ability to predict the 30-day mortality risk in patients following ICH surgery. It also explored the role of postoperative pH, lactate, activated partial thromboplastin time (APTT), and C-reactive protein (CRP) in patient prognosis. Compared to existing research, our study demonstrated substantial improvements in both model performance and interpretability.

Recent years have seen significant progress in the prognostic modeling of ICH (19, 20). For instance, Chen et al. (21) and Song et al. (22) have developed algorithms to predict hematoma expansion and functional outcomes, respectively. Regarding mortality prediction, Chen et al. recently utilized a deep learning survival model to estimate outcomes based on initial computed tomography scans, highlighting the potential of automated radiologic assessment (23). Our study specifically focuses on the survival of postoperative ICH patients. Compared with models relying on initial structural imaging, our approach integrates dynamic perioperative data—including intraoperative events and postoperative biomarkers—enabling more granular risk stratification during the critical early postoperative phase. Furthermore, SHAP interpretability helps clinicians identify key prognostic predictors, facilitating the formulation of targeted therapeutic interventions.

Among the predictive models in this study, the XGBoost model demonstrated superior discrimination compared with SVM and LR. This performance advantage likely stems from the ability of gradient boosting frameworks to effectively capture the complex, non-linear interactions and iteratively correct prediction errors inherent in heterogeneous perioperative data. In contrast, traditional linear models or kernel-based methods often lack the robustness required to manage the high degree of heterogeneity characteristic of such clinical tabular datasets.

In our study, we identified pivotal immunometabolic biomarkers, specifically postoperative pH and lactate, as independent predictors of prognosis in ICH patients. Lactate accumulation is typically a result of anaerobic metabolism, which occurs under conditions of localized hypoxia (24). During hypoxia, cells increase lactate production through anaerobic glycolysis, leading to a decrease in extracellular PH. This acidic environment not only exacerbates neuronal death (25) and tissue necrosis (26) but also activates calcium-dependent enzymes such as proteases and phospholipase A2, thereby accelerating neuronal damage (27). Additionally, acidic conditions promote the release of pro-inflammatory cytokines, including IL-6 and TNF-α, further intensifying systemic inflammatory responses. These mechanisms may explain the observations by Wu et al. (28), which noted an association between low pH levels and increased mortality and neurological deterioration in ICH patients.

In critically ill patients with intracerebral hemorrhage, abnormalities in lactate and pH typically manifest as indicators of systemic shock or compromised tissue perfusion. Consequently, for patients exhibiting lactate abnormalities, the primary clinical imperative is to address the underlying reasons, such as low cardiac output or sepsis. Beyond its established role as hemodynamic indicator, lactate is also increasingly recognized as active biological signals that modulate the immune system. Elevated lactate levels can promote the activation of myeloid-derived suppressor cells (MDSCs) and regulatory T cells (Tregs), fostering an immunosuppressive state that may hinder neural repair (29). Therefore, while standard care focuses on hemodynamic stability, strategies that also address these immunometabolic pathways could potentially offer additional benefits for neurological recovery.

Specific immunotherapeutic strategies include the use of metabolic modulators to reduce lactate production and accumulation. For example, agents such as metformin and diacetyl resveratrol have been shown to lower lactate levels by influencing mitochondrial metabolism, thereby mitigating the acidic environment in hypoxic tissues (30). Furthermore, experimental studies are exploring the activation of lactate transporters, such as monocarboxylate transporters MCT1 and MCT4, to enhance lactate efflux from cells, alleviating local lactate buildup and its associated acidic effects (31). Through these approaches, controlling lactate levels not only aids in pH balance but also plays a critical role in modulating the immune system, thereby reducing inflammation and promoting tissue repair and neurological recovery.

APTT, another significant factor identified in our study, plays a crucial role in predicting postoperative outcomes in ICH patients (32). As a measure of intrinsic coagulation pathway activity, prolonged APTT often indicates reduced clotting factor activity or elevated levels of anticoagulants. Li et al. (33) reported that prolonged APTT is strongly associated with higher risks of postoperative hemorrhage and mortality, particularly in critically ill patients requiring intensive coagulation management. In severe ICH cases, extended APTT not only reflects coagulation disturbances but also signals an increased risk of recurrent or expanding hemorrhages, potentially leading to further complications like cerebral edema and elevated intracranial pressure. By including APTT in our predictive model, we improved the precision of hemorrhage risk assessment, offering valuable guidance for optimizing anticoagulant and antiplatelet therapy.

C-reactive protein, widely recognized as an acute-phase reactant, is frequently used to assess inflammation and tissue damage (34). Our study found that elevated postoperative CRP levels were strongly correlated with 30-day mortality in ICH patients. Elevated CRP levels not only indicate acute inflammatory responses but also signify a prolonged inflammatory state contributing to brain tissue damage. Wang et al. (35) found that increased CRP levels were linked to larger hematoma volumes and poorer neurological recovery in ICH patients. This suggests that CRP not only serves as a sensitive marker for monitoring postoperative inflammation but also plays a critical role in guiding anti-inflammatory therapy (36, 37).

Our study does have some limitations. Firstly, the data were sourced from a public database, which inevitably led to issues with missing variables. To address this, we employed MICE techniques to minimize bias and ensure the robustness of our analysis. However, traditional neuro-critical metrics (such as GCS and hematoma volume) were unavailable in the public dataset. These indicators are not only widely recognized as robust independent predictors of prognosis, but are also essential components of the standard ICH score. Consequently, their absence precludes a direct performance comparison between our machine learning model and traditional clinical scores. Secondly, the dataset was limited to Korean patients, which may restrict the generalizability of our model to other populations. Finally, our mortality prediction model relies primarily on data from the first 24 h following ICU admission, potentially overlooking late-phase events that could affect outcomes. In the future, conducting a prospective multi-center cohort study to specifically incorporate these key neurological indicators into the model for further analysis is essential. Additionally, incorporating dynamic patient data, such as treatment responses and postoperative complications, will enhance the model’s predictive accuracy under changing clinical conditions.

## 5 Conclusion

This study established an interpretable XGBoost model utilizing perioperative data to accurately predict 30-day mortality following ICH surgery. By leveraging SHAP analysis, we identified postoperative pH and lactate as pivotal immunometabolic biomarkers that, alongside APTT and CRP, serve as independent prognostic factors.

## Data Availability

The raw data supporting the conclusions of this article will be made available by the authors, without undue reservation.
